# The Association Between Differing Grip Strength Measures and Mortality and Cerebrovascular Event in Older Adults: National Health and Aging Trends Study

**DOI:** 10.3389/fphys.2018.01871

**Published:** 2019-01-07

**Authors:** Daniel G. Whitney, Mark D. Peterson

**Affiliations:** Department of Physical Medicine and Rehabilitation, University of Michigan, Ann Arbor, MI, United States

**Keywords:** grip strength, normalized grip strength, mortality, cerebrovascular event, elderly, National Health and Aging Trends Study

## Abstract

The purpose of this study was to compare the predictive capacity of different post-processing methods of hand grip strength (GS) for mortality and incident cerebrovascular events in older adults. A sample of 4,143 participants aged 65 years and older was included from the National Health and Aging Trends Study (NHATS) and followed for 6 years. GS measures included baseline (i.e., round 1) (1) absolute GS, (2) GS divided by body mass (NGS_mass_), and (3) GS divided by body mass index (NGS_BMI_), as well as (4) change in absolute GS from round 1 to round 2 (GS_1-2_). Cox proportional hazards regression models were used to examine the association between sex- and age group-specific tertiles of GS measures (weak, moderate-strength, strong) with mortality (*n* = 641) and incident cerebrovascular events (*n* = 329). Absolute GS (hazard ratio [HR] = 1.83; 95% confidence interval [CI] = 1.51–2.22), NGS_mass_ (HR = 1.46; 95% CI = 1.21–1.76), and NGS_BMI_ (HR = 1.50; 95% CI = 1.24–1.82) were each associated with mortality among weak participants, but not GS_1-2_ (HR = 1.10; 95% CI = 0.99–1.46). NGS_mass_ (HR = 1.54; 95% CI = 1.19–2.01) and NGS_BMI_ (HR = 1.37; 95% CI = 1.06–1.79) were both associated with incident cerebrovascular event among weak participants, but not absolute GS (HR = 1.12; 95% CI = 0.86–1.47) or GS_1-2_ (HR = 1.11; 95% CI = 0.85–1.44). Absolute GS, NGS_mass_, and NGS_BMI_ were each associated with mortality, whereas only NGS_mass_ and NGS_BMI_ were associated with cerebrovascular event. These findings suggest that different post-processing methods of GS may have differing predictive capacity in the elderly depending on the outcome of interest; however, since NGS measures were associated with both mortality and cerebrovascular events, they may be considered advantageous for screening in older adults.

## Introduction

Muscle strength capacity is a primary determinant of many functional aspects of daily living in older adults, including physical function, cardiometabolic health, and psychosocial wellbeing. The preservation of muscle strength with advancing age, through physical activity and exercise, is an important determinant of disease prevention and longevity (McGrath et al., 2018a). Hand grip strength (GS) is a reliable, inexpensive, and easily utilized surrogate of muscle strength (Peolsson et al., 2001; Savva et al., 2014), and has shown validity and reliability between different devices (Chkeir et al., 2012). Given that GS is highly associated with other measures of muscle strength (e.g., lower extremity strength capacity), it may be considered a valid proxy indicator of overall strength capacity (Cooper et al., 2013). Muscle weakness, as determined by GS assessment, is associated with increased risk of functional disabilities (McGrath et al., 2017b, 2018c), fracture (Dixon et al., 2005), cardiometabolic disease (Peterson et al., 2016a,b,d, 2017; McGrath et al., 2017c), musculoskeletal morbidities (Rikkonen et al., 2012; McGrath et al., 2017a), and early mortality (Leong et al., 2015; Peterson et al., 2016c; Oksuzyan et al., 2017; Celis-Morales et al., 2018). Moreover, statistical modeling of GS significantly improves prediction of morbidity and mortality beyond established office based risk scores (Celis-Morales et al., 2018), and is a stronger predictor of all-cause mortality than even systolic blood pressure (Leong et al., 2015).

Representatives from a variety of institutions participating in the Foundation for the National Institutes of Health (FNIH) Sarcopenia Project concluded that GS should be utilized to assess muscle weakness in the clinical setting (Studenski et al., 2014). There has been debate about the optimal methods for modeling GS in statistical prediction across health outcomes and populations. Most studies investigate absolute GS in an attempt to simplify the interpretation of findings in singular units; whereas we and others have preferred the use of normalizing GS (NGS) by incorporating body composition measures relative to GS (Lawman et al., 2016; Peterson et al., 2016a,b,c, 2017; McGrath et al., 2017a,b,c, 2018c). The FNIH Sarcopenia Project found that muscle weakness defined by GS normalized to body mass index was a stronger predictor of mobility impairment than absolute GS (McLean et al., 2014). Other post-processing techniques that are easily interpretable and computed in a clinical setting include normalizing GS to body mass (Peterson et al., 2016a,b) or assessing change in GS over time (Sirola et al., 2006; Karvonen-Gutierrez et al., 2018). Identifying which post-processing methods of GS is the strongest predictor of clinically important outcomes among older adults will provide clinicians better predictive options for evaluating muscle weakness in the elderly population. This has important implications for longitudinal monitoring or evaluating the efficacy of exercise interventions aimed at mitigating adverse health outcomes in the elderly. Accordingly, the purpose of this study was to determine which of the most common and easily utilized post-processing methods of GS (i.e., absolute GS, normalized GS, change in GS) was the strongest predictor of mortality and incident cerebrovascular events (myocardial infarction or stroke) in a sample of Medicare beneficiaries aged 65 and older.

## Materials and Methods

### Participants

Data were from the National Health and Aging Trends Study (NHATS). NHATS utilized a multistage survey design, sampling >8,000 Medicare beneficiaries aged 65+ with an annual face-to-face interview conducted by trained study personnel. Non-Hispanic Blacks and those aged 90+ were oversampled. NHATS started in 2011 and subjects were assessed each year for a total of six rounds. Response rates were 71% at baseline. Additional information pertaining to NHATS study design, methodology, and survey instruments is available from https://www.nhats.org/. The NHATS study protocol was approved by The Johns Hopkins University Institutional Review Board.

Of the 8,245 participants at baseline, 4,102 participants were excluded from the analyses because they dropped out of the study, were unable to answer survey questions on their own, had dementia, or had incomplete data for baseline GS, baseline body mass, baseline height, round 2 GS, or survival. Survey weights were not applied because the purpose of this study was to compare the different post-processing methods of GS for mortality and incident cerebrovascular events. Therefore, the sample is the same for each of the outcome variables.

### Outcome Variables

The participant’s death was reported to the study personnel by informants during attempts to contact the participant for their annual interview. Since inclusion criteria required data for round 2 GS, survival time was computed as the annual rate for living and deceased participants from round 1 (alive) to rounds 3–6.

An incident cerebrovascular event was determined if participants reported myocardial infarction or stroke on the basis of an affirmative response to: “Please tell me if a doctor ever told you that you had [a heart attack or myocardial infarction/a stroke]?” Participants were excluded from analyses with cerebrovascular event as the outcome variable if they reported a cerebrovascular event at round 1. Myocardial infarction and stroke were combined into 1 category because of the low number of individuals who experienced either event that also met inclusion criteria.

### Grip Strength Variables

The NHATS measured absolute GS (in kg) using a digital, adjustable hand dynamometer (Jamar Plus) in those that did not have surgery or flare up of pain in both hands or wrists, or have surgery in the arms or shoulders within the last 3 months. Participants were asked to squeeze the dynamometer as hard as they could with their arm at their side and elbow bent at 90 degrees. GS was measured twice and the highest value was used for this investigation. Height and body mass were self-reported. Body mass index (BMI) was calculated as follows: body mass (kg)/height (m)^2^. Four GS measures were computed: (1) baseline absolute GS; (2) baseline absolute GS divided by body mass (NGS_mass_); (3) baseline absolute GS divided by BMI (NGS_BMI_); and (4) percent change in GS from round 1 to round 2 (GS_1-2_).

### Demographic Variables

Age and sex were available for all participants. Age was categorized into the following groups: 65–74, 75–84, and 85+ years. Weight status was determined by BMI and separated into the following categories: underweight (<18.5 kg/m^2^); normal weight (18.5–24.9 kg/m^2^); overweight (25.0–29.9 kg/m^2^); and obese (≥30.0 kg/m^2^).

### Statistical Analysis

Descriptive characteristics and GS measures were summarized as means ± SD or frequency (percentage). For each GS measure, sex- and age group-specific tertiles were created to categorize participants into the following muscle strength capacity groups: weak, moderate-strength, and strong. This method allowed for the comparative predictive assessment of GS for each of the outcomes of interest without introducing the confounding effects of age and sex. Unadjusted Cox proportional hazards regression models were used to examine the association between each transformed GS measure and mortality and incident cerebrovascular events. Participants were right censored at round 6 if they were alive (when modeling for mortality) or had no cerebrovascular events (when modeling for incident cerebrovascular events). Since GS measures were standardized using sex and age groups, the hazard ratios (HR) and 95% confidence intervals (CI) were examined to determine which sex- and age-adjusted GS measures were the strongest predictor of each outcome. All statistical analyses were performed using SAS 9.4 (SAS Institute, Cary, NC, United States).

## Results

Descriptive characteristics and GSs of study participants in the entire sample (*n* = 4,143) and by sex (45.1% male) are presented in Table 1. Over the 6 rounds, 641 participants were reported to be deceased (15.5%). Of those without a reported cerebrovascular event at round 1 (*n* = 3,309), 329 had acquired a cerebrovascular event (9.9%).

**Table 1:** Baseline descriptive characteristics and grip strength (GS) measures of the participants.

	Overall (*n* = 4,143)	Men (*n* = 1,869)	Women (*n* = 2,274)
**Age, n (%)**			
65–74	1,817 (43.9)	872 (46.7)	945 (41.6)
75–84	1,649 (39.8)	727 (38.9)	922 (40.5)
85+	677 (16.3)	270 (14.4)	407 (17.9)
**Weight status, n (%)**			
Underweight	76 (1.8)	19 (1.0)	57 (2.5)
Normal weight	1,366 (33.0)	553 (29.6)	813 (35.8)
Overweight	1,571 (37.9)	833 (44.6)	738 (32.4)
Obese	1,130 (27.3)	464 (24.8)	666 (29.3)
**GS**			
Mean ± SD	27.2 ± 10.5	35.0 ± 9.6	20.9 ± 5.9
NGS_mass_, mean ± SD	0.35 ± 0.12	0.42 ± 0.12	0.30 ± 0.10
NGS_BMI_, mean ± SD	1.02 ± 0.41	1.30 ± 0.39	0.79 ± 0.26
GS_1-2_, mean ± SD	-0.51 ± 22.5	-0.81 ± 22.3	-0.26 ± 22.7
Deceased, n	641	330	311
Cerebrovascular event, n	329	156	173


*NGS, normalized GS; NGS_*mass*_, GS divided by body mass; NGS_*BMI*_, GS divided by body mass index; GS_*1*-*2*_, percent change of GS from round 1 to round 2 (1 year).*

Table 2 shows the results of the Cox regression models for the association between sex- and age-specific GS tertiles (reference: strong participants [highest tertile]) and mortality. For weak participants (lowest tertile), GS had the largest HR with mortality (HR = 1.83; 95% CI = 1.51–2.22), followed by NGS_BMI_ (HR = 1.50; 95% CI = 1.24–1.82) and NGS_mass_ (HR = 1.46; 95% CI = 1.21–1.76). GS_1-2_ was not significantly associated with mortality for weak participants (HR = 1.20; 95% CI = 0.99–1.46). For moderate-strength participants (middle tertile), only GS was significantly associated with mortality (HR = 1.42; 95% CI = 1.16–1.73).

**Table 2:** Cox proportional hazards regression for the association between sex- and age group-specific tertiles for grip strength (GS) measures (reference group: strong tertile) with mortality (*n* = 4,143) and cerebrovascular event (*n* = 3,309).

	Mortality	Cerebrovascular event

Exposure	HR (95% CI)	HR (95% CI)
**GS**		
Weak	**1.83 (1.51, 2.22)**	1.12 (0.86, 1.47)
Moderate-strength	**1.42 (1.16, 1.73)**	1.12 (0.87, 1.44)
**NGS_mass_**		
Weak	**1.46 (1.21, 1.76)**	**1.54 (1.19, 2.01)**
Moderate-strength	1.03 (0.84, 1.26)	1.06 (0.81, 1.40)
**NGS_BMI_**		
Weak	**1.50 (1.24, 1.82)**	**1.37 (1.06, 1.79)**
Moderate-strength	1.07 (0.87, 1.31)	1.05 (0.80, 1.37)
**GS_1-2_**		
Weak	1.20 (0.99, 1.46)	1.11 (0.85, 1.44)
Moderate-strength	1.14 (0.94, 1.38)	1.03 (0.79, 1.34)


*HR, hazard ratio; CI, confidence interval; NGS, normalized grip strength; NGS_*mass*_, absolute grip strength divided by body mass; NGS_*BMI*_, absolute grip strength divided by body mass index; GS_*1*-*2*_, percent change of grip strength from round 1 to round 2. Significant HRs are bolded.*

Table 2 shows the results of the Cox regression models for the association between sex- and age-specific GS tertiles (reference: strong participants) and incident cerebrovascular events. For weak participants, NGS_mass_ had the largest HR with cerebrovascular event (HR = 1.54; 95% CI = 1.19–2.01), followed by NGS_BMI_ (HR = 1.37; 95% CI = 1.06–1.79). GS (HR = 1.12; 95% CI = 0.86–1.47) and GS_1-2_ (HR = 1.11; 95% CI = 0.85–1.44) were not significantly associated with a cerebrovascular event. For moderate-strength participants, none of the predictors were significantly associated with a cerebrovascular event (HR = 1.03–1.12; all *p* > 0.05).

## Discussion

The primary findings of this study were that both GS and NGS measures were significant predictors of mortality in older adults, and that NGS measures were significant predictors of incident cerebrovascular events. For predicting mortality, GS had a higher HR than NGS or change in GS; whereas NGS measures were stronger predictors of cerebrovascular events than GS or changes in GS. These findings suggest that different post-processing methods of GS may have differing predictive capacities in the elderly depending on the outcome of interest; however, since NGS was robustly associated with both mortality and cerebrovascular events, it may be considered as a viable standalone tool for screening in older adults. Moreover, considering the well-established role of exercise and physical activity on muscle strength, body composition, and mitigating adverse health outcomes, NGS may serve as a better proxy than absolute GS for determining efficacy of exercise interventions because it encompasses both muscle strength and body composition.

The findings that absolute and NGS measures were associated with mortality and cerebrovascular event are consistent with previous reports (Leong et al., 2015; Peterson et al., 2016c; Oksuzyan et al., 2017; Celis-Morales et al., 2018). The finding that change in GS was not associated with adverse health outcomes is consistent with another report (Karvonen-Gutierrez et al., 2018). While it seems intuitive that a higher rate of strength decline would correspond to a higher rate of acquiring adverse health outcomes, our methodology was limited in adequately addressing this notion. In the current investigation, we used a time interval of 1 year to assess strength change, which may not have been long enough to capture greater strength declines across advancing age. Moreover, our sample included adults 65 years and older. The rate of strength decline from young- or middle-age may be more predictive of later functioning and health outcomes in the elder years.

The difference in associations between absolute GS versus NGS measures with mortality and cerebrovascular events may reflect the influence of body composition or the role of obesity. Myint et al. (2014) found that measures of body composition (BMI, body fat percent, and waist-to-hip ratio) were stronger predictors of incident cardiovascular disease than mortality in middle- and older-age adults. Therefore, by incorporating body mass or BMI, NGS may be a superior predictor for cerebrovascular events than for mortality, as it encompasses important constituents (i.e., body composition) for cerebrovascular function.

Another potential explanation for the unique associations found for absolute GS vs. NGS measures is the so-called “obesity paradox,” where there is lower mortality in those with cardiovascular disease who are obese compared to non-obese (Curtis et al., 2005; Angeras et al., 2013; Flegal et al., 2013), but not in those who are morbidly obese (Angeras et al., 2013). In the publically available NHATS dataset, mortality information is denoted as “deceased” or not, thus providing a “catch-all” cause of mortality. Further, in the current investigation, all participants who reported a cerebrovascular event were alive in the same round. Therefore, since absolute GS is a general measure of muscle strength capacity, it may capture the wider and non-specific construct of all-cause mortality and reflect the obesity paradox, i.e., those with greater BMIs may have greater absolute GSs (Lawman et al., 2016). On the other hand, GS normalized to body composition may be more specific to cardiometabolic-related morbidity and mortality, i.e., those with greater body masses or BMIs relative to GS may reflect poor cerebrovascular and metabolic health profiles (Lawman et al., 2016). Unfortunately, we were unable to determine how the different post-processing methods of GS were associated with specific causes of mortality.

The association between muscle weakness and adverse health outcomes is likely driven in part by poor physical functioning (McGrath et al., 2018b). While muscle weakness is inversely associated with physical functioning (Henriksen et al., 2012; Ryder et al., 2013), normalized strength is more strongly associated with physical functioning than absolute strength (Schiller et al., 2000; Henriksen et al., 2012). The caveat in examining absolute GS is that individuals with a high body mass or BMI likely have a higher GS relative to their physical functioning ability. Therefore, normalized strength capacity may provide a better indicator of the ability for that individual to maneuver his/her body through space and perform physical activities. Interestingly, when we examined the strength profiles of the obese participants who were classified as “strong” according to absolute GS (upper GS tertile), nearly 80% were considered “moderate-strength” or “weak” according to NGS measures. These findings highlight the potential benefits of using NGS in evaluating muscle strength capacity in the elderly.

There are other limitations that need to be discussed. First, height and weight were self-reported, which may have influenced measures adjusting for body composition. Stommel and Schoenborn (2009) found that misclassification of weight status by self-report height and weight to determine BMI was more pronounced on the extreme ends, including underweight and obese. However, deviations of BMI values were modest, with the majority (56%) of misclassifications having self-reported BMI values within one-unit interval of their measured BMI. Second, we did not adjust models for sociodemographics, socioeconomics, or morbidities. Whether the difference in associations among the GSs with outcomes are mediated uniquely by confounding variables is unknown and requires future investigation.

## Conclusion

In conclusion, NGS measures were significantly associated with both mortality and incident cerebrovascular event, whereas absolute GS was only significantly associated with mortality. Changes in GS were not significantly associated with mortality or incident cerebrovascular event; however, the lack of association may have been due to a short follow up period. These findings are important as they provide evidence of unique associations between clinically important aging outcomes with a variety of commonly used post-processing methods of GS that can be easily utilized in a clinical setting.

## Author Contributions

DW and MP designed the study and approved the final manuscript. DW contributed to data ascertainment, data analysis, and prepared the manuscript.

## Conflict of Interest Statement

The authors declare that the research was conducted in the absence of any commercial or financial relationships that could be construed as a potential conflict of interest.
